# Phenotypic and Genetic Characteristics in a Cohort of Patients with Usher Genes

**DOI:** 10.3390/genes13081423

**Published:** 2022-08-10

**Authors:** Helena M. Feenstra, Saoud Al-Khuzaei, Mital Shah, Suzanne Broadgate, Morag Shanks, Archith Kamath, Jing Yu, Jasleen K. Jolly, Robert E. MacLaren, Penny Clouston, Stephanie Halford, Susan M. Downes

**Affiliations:** 1 Nuffield Laboratory of Ophthalmology, Nuffield Department of Clinical Neurosciences, University of Oxford, Oxford OX3 9DU, UK; 2 Oxford Eye Hospital, Oxford University Hospitals NHS Foundation Trust, Oxford OX3 9DU, UK; 3 Oxford Medical Genetics Laboratories, Oxford University Hospitals NHS Foundation Trust, Oxford OX3 7LE, UK; 4 Wellcome Centre for Human Genetics, NIHR Oxford Biomedical Research Centre, University of Oxford, Oxford OX3 7BN, UK; 5 Vision and Eye Research Institute, Anglia Ruskin University, Cambridge CB1 1PT, UK

**Keywords:** usher syndrome types 1, 2, 3, *USH2A*, retinitis pigmentosa, syndromic retinitis pigmentosa, non-syndromic autosomal recessive retinitis pigmentosa (NS-ARRP)

## Abstract

Background: This study aimed to compare phenotype–genotype correlation in patients with Usher syndrome (USH) to those with autosomal recessive retinitis pigmentosa (NS-ARRP) caused by genes associated with Usher syndrome. Methods: Case notes of patients with USH or NS-ARRP and a molecularly confirmed diagnosis in genes associated with Usher syndrome were reviewed. Phenotypic information, including the age of ocular symptoms, hearing impairment, visual acuity, Goldmann visual fields, fundus autofluorescence (FAF) imaging and spectral domain optical coherence tomography (OCT) imaging, was reviewed. The patients were divided into three genotype groups based on variant severity for genotype-phenotype correlations. Results: 39 patients with Usher syndrome and 33 patients with NS-ARRP and a molecular diagnosis in an Usher syndrome-related gene were identified. In the 39 patients diagnosed with Usher syndrome, a molecular diagnosis was confirmed as follows: *USH2A* (28), *MYO7A* (4), *CDH23* (2), *USH1C* (2), *GPR98/VLGR1* (2) and *PCDH15* (1). All 33 patients with NS-ARRP had variants in *USH2A*. Further analysis was performed on the patients with *USH2A* variants. USH2A patients with syndromic features had an earlier mean age of symptom onset (17.9 vs. 31.7 years, *p* < 0.001), had more advanced changes on FAF imaging (*p* = 0.040) and were more likely to have cystoid macular oedema (*p* = 0.021) when compared to *USH2A* patients presenting with non-syndromic NS-ARRP. Self-reported late-onset hearing loss was identified in 33.3% of patients with NS-ARRP. Having a syndromic phenotype was associated with more severe *USH2A* variants (*p* < 0.001). Eighteen novel variants in genes associated with Usher syndrome were identified in this cohort. Conclusions: Patients with Usher syndrome, whatever the associated gene in this cohort, tended to have an earlier onset of retinal disease (other than GPR98/VLGR1) when compared to patients presenting with NS-ARRP. Analysis of genetic variants in *USH2A*, the commonest gene in our cohort, showed that patients with a more severe genotype were more likely to be diagnosed with USH compared to NS-ARRP. *USH2A* patients with syndromic features have an earlier onset of symptoms and more severe features on FAF and OCT imaging. However, a third of patients diagnosed with NS-ARRP developed later onset hearing loss. Eighteen novel variants in genes associated with Usher syndrome were identified in this cohort, thus expanding the genetic spectrum of known pathogenic variants. An accurate molecular diagnosis is important for diagnosis and prognosis and has become particularly relevant with the advent of potential therapies for Usher-related gene

## 1. Introduction

Usher syndrome (USH) is an autosomal recessive disorder characterised by retinitis pigmentosa (RP), sensorineural hearing loss, and in some cases, vestibular dysfunction. USH is clinically and genetically heterogeneous and is the most common cause of deafness and blindness, with an estimated prevalence of 3.5–16.6 in 100,000 [1]. However, not all patients have syndromic features.

Clinically, USH is categorised into three groups: Usher syndrome type 1 (USH1), which is the most severe form and is characterised by severe to profound congenital hearing impairment, prepubertal onset of RP, and vestibular dysfunction; Usher syndrome type 2 (USH2), which is characterised by congenital moderate to severe hearing impairment, the onset of RP in the first or second decade of life, and normal vestibular function; and Usher syndrome type 3 (USH3) which is defined by a congenital or early onset of progressive hearing impairment, whereas the onset and severity of RP, as well as the vestibular function, are highly variable [2,3,4].

Thus far, USH has been associated with mutations in 14 different genes. These genes primarily encode proteins located in the region of the connecting cilium of retinal photoreceptors and hair cells of the inner ear. For USH1, seven genes have been identified to date: *MYO7A* (myosin VIIA; OMIM 276903), *USH1C* (*USH1 protein network component; OMIM* 605242), *PCDH15* (protocadherin 15; *OMIM* 605514), *USH1G* (*USH1 protein network component sans; OMIM* 607696), *CDH23* (cadherin 23*; OMIM* 605516), *CIB2* (calcium and integrin binding protein 2; OMIM 605564) [4], and *ESPN* (Espin; OMIM 606351). USH2 has been associated with three genes: *USH2A* (*usherin*; *OMIM* 608400), *ADGRV1/GPR98* (adhesion G protein-coupled receptor V1*; OMIM* 602851), and *DFNB31/WHRN* (*whirlin*; *OMIM* 607928) [4]. Variants in *USH2A* account for over half of all USH cases [5] and have been reported to account for up to 79% of USH2 patients [4]. *CLRN1* (clarin 1; OMIM 606397) has been associated with USH3. A further category called atypical USH has been associated with variants in the *ABHD12* (abhydrolase domain containing 12; OMIM 613599), *ARSG* (arylsulfatase G; OMIM 618144), *CEP78* (*centrosomal protein 78;* OMIM 617236) and *HARS* (histidyl-tRNA synthetase; OMIM 142810). However, *ABHD12* is not expressed in the connecting cilium. Finally, two modifier genes have been described in association with USH; *PDZD7* (PDZ domain containing 7; OMIM 612971) and *CEP250* (centrosomal protein 250; OMIM 609689). These modifier genes encode ciliary proteins and have been reported to cause severe retinal involvement due to an additive effect [6,7,8].

Variants in genes associated with USH have also been identified in patients with non-syndromic autosomal recessive retinitis pigmentosa (NS-ARRP), who present with RP and do not have any extra-ocular signs or symptoms. Variants in the Usherin (*USH2A*) gene are frequently identified in NS-ARRP patients and have been reported to account for 19–25% of NS-ARRP [9,10]. However, some studies have found that patients with NS-ARRP secondary to variants in *USH2A* may develop late-onset hearing loss [1,9,11]. The c.2299delG, p.(Glu767SerfsTer21) variant in exon 13 of the *USH2A* gene has been reported to account for 16% of alleles in patients with *USH2A* retinopathy [3,9]. Genotype-phenotype correlation and confirmation of the pathogenicity of the variants within exon 13 will become increasingly more important as therapeutic interventions are currently being investigated in this exon. The genotype-phenotype correlation in *USH2A* retinopathy is currently being investigated in the rate of progression in *USH2A*-related retinal degeneration (RUSH2A) natural history study, which includes 127 internationally recruited patients [12].

Different disease mechanisms have been proposed to explain why *USH2A* mutations lead to USH in some patients and NS-ARRP in others. Lenassi et al. [9] have proposed a model of ‘retinal disease-specific’ *USH2A* alleles (i.e., alleles associated with retinal degeneration and no hearing loss in childhood). The presence of at least one such allele in a patient with *USH2A-*related retinal degeneration may result in relative preservation of hearing. The c.2766G > T p.(Cys759Phe) variant is the second most common *USH2A* variant in the Leiden Open Variant Database (LOVD www.LOVD.nl/USH2A) and has previously been associated with RP without hearing impairment [9,11,13]. In addition to c.2766G > T, Lenassi et al. have described five other likely retinal disease-specific variants: c.2802T>G p.(Cys934Trp); c.10073G > A p.(Cys3358Tyr); c.11156G > A p.(Arg3719His); c.12295-3T > A and c.12575G>A p.(Arg4192His) [9]. It is difficult to ascertain the retinal disease-specific nature of these alleles in the absence of data from other cohorts of *USH2A*-related NS-ARRP. Indeed Pierrache et al. [3] identified the c.2276G>T variant in six compound heterozygous patients with syndromic features. The LOVD (accessed on 4 July 2022) also reports several patients with Usher syndrome type 2A with hearing loss in patients carrying the c.2276G > T and c.11156G > A variants. This led Pierrache et al. [3] to propose that normal cochlear development depends on the presence of at least 1 functional copy of the *USH2A* protein. More recently, it has been proposed that severe variants that result in a truncated protein might be associated with syndromic disease in patients with *USH2A* retinopathy that are also associated with earlier disease onset [3,14,15], whilst missense variants have been reported to occur in non-syndromic RP [12]. The RUSH2A natural history study has also found that patients with USH syndrome had more severe visual field loss compared to patients with NS-ARRP secondary to variants in *USH2A* [16].

These studies highlight the need for phenotype–genotype correlation studies to clarify whether specific variants or types of variants are associated with NS-ARRP or USH. This is important for patient management, genetic counselling, potential therapeutic interventions, and for providing prognostic information to patients with USH or NS-ARRP caused by variants in genes associated with USH.

## 2. Materials and Methods

All patients from a single centre diagnosed with USH or NS-ARRP associated with pathogenic variants in genes associated with USH were identified retrospectively between July 2013–October 2021. This study adhered to the tenets of the Declaration of Helsinki and was approved by the Essex 2 Research Ethics Committee (reference 08/H0302/96). Informed consent was obtained from all participants.

### 2.1. Clinical Phenotype

Clinical data collected from the patient’s first visit included: best corrected visual acuity (BCVA), colour fundus photography, short-wavelength fundus autofluorescence (FAF; Spectralis, Heidelberg Engineering, Heidelberg, Germany), spectral domain–optical coherence tomography (SD–OCT; Spectralis, Heidelberg Engineering, Heidelberg, Germany), Goldmann visual field (GVF), and information regarding the subjective presence of any hearing impairment or use of hearing aids.

Best corrected visual acuity was recorded on a Snellen chart and was converted to LogMAR for the purposes of statistical analysis [17]. Visual acuity of counting fingers or less was not included in the statistical analysis due to a lack of information at what distance this had been recorded. Goldmann perimetry was conducted using I4e, III4e and V4e white kinetic stimuli against a standard background of 31.5 apostilbs. The GVF were graded as to whether there were >10 degrees or <10 degrees remaining at the time of baseline examination. Electroretinography was performed in accordance with the standards of the International Society of Electrophysiology of Vision (ISCEV) using DTL fibre electrodes and an impedance < 5 kOhms in pupils dilated with 1% tropicamide [18]. Information about self-reported hearing loss, the use of hearing aids and vestibular impairment were retrieved from the case notes.

**Figure 1:**
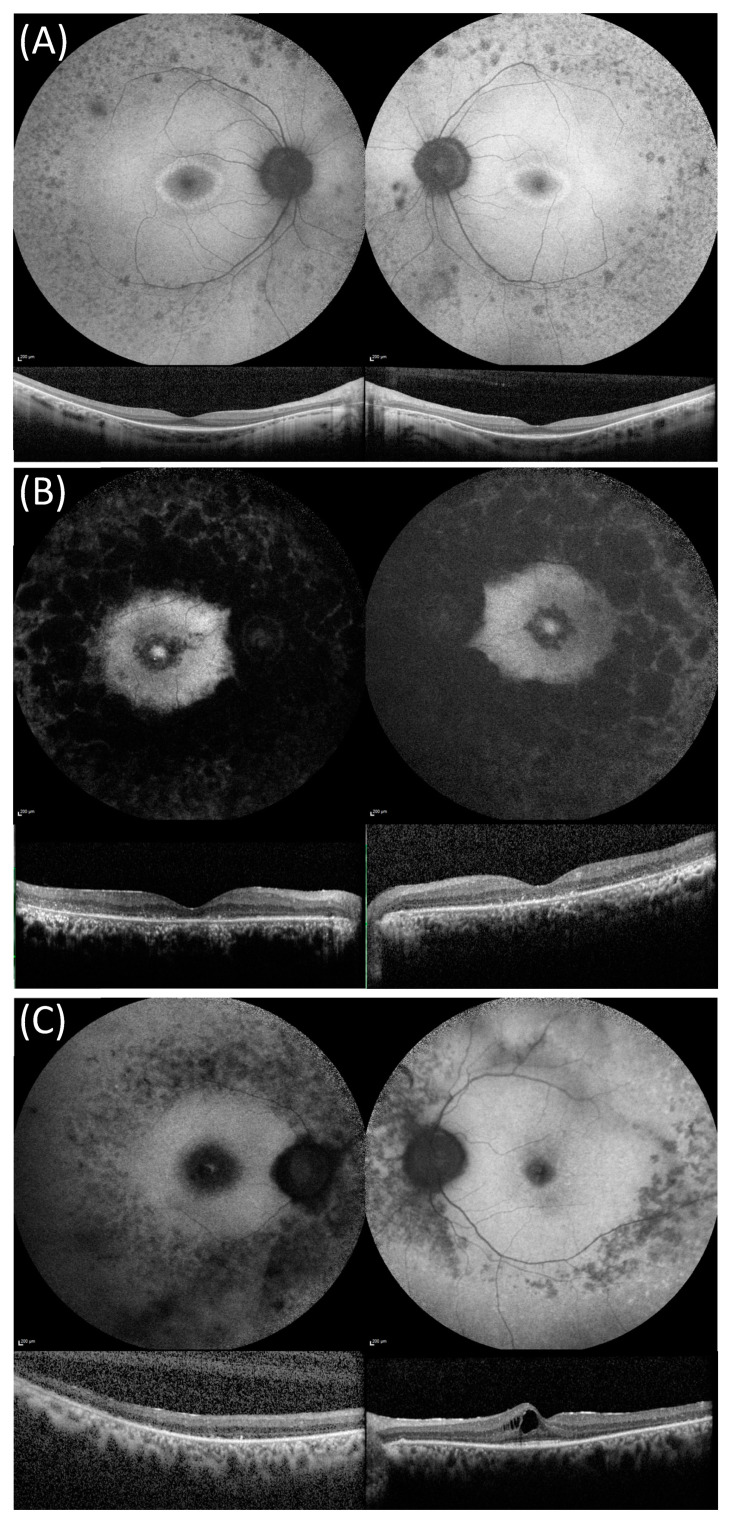
Fundus autofluorescence (FAF) and optical coherence tomography (OCT) images in three compound heterozygous patients from our cohort showing the disease stages described by Fakin et al. [19] for *USH2A* variants. (**A**) FAF and OCT imaging of patient 50 with non-syndromic autosomal recessive retinitis pigmentosa phenotype that was compound heterozygous for *USH2A* variants c.2276G > T, p.(Cys759Phe) and c.11875_11876delCA. FAF images show a ring of raised AF surrounding the foveal region and patches of decreased AF in the mid-peripheral retina. OCT images showing central retinal preservation. (**B**) FAF and OCT imaging of patient 35 with USH that was compound heterozygous for *USH2A* c.9469C > T p.(Gln3157*) and the novel c.10586-1_10595delins13 variant. FAF images show an increased patch of raised AF surrounded by a region of reduced AF and also raised macular AF, which is surrounded by large patches of decreased AF that extend into and beyond the mid-peripheral retina. OCT images show loss of outer retinal layers. (**C**) FAF and OCT imaging for patient 12 with USH that was compound heterozygous for c.7932G-A, p.(Trp2644*), c.13331C > T p.(Pro4444Leu), and the variant of unknown significance c.6364G > T p.(Ala2122Ser). FAF images showed decreased foveal AF and decreased AF in the mid-peripheral retina. OCT images show a significant outer-retinal loss in the right eye and cystoid macular oedema in the left eye.

**Figure 2:**
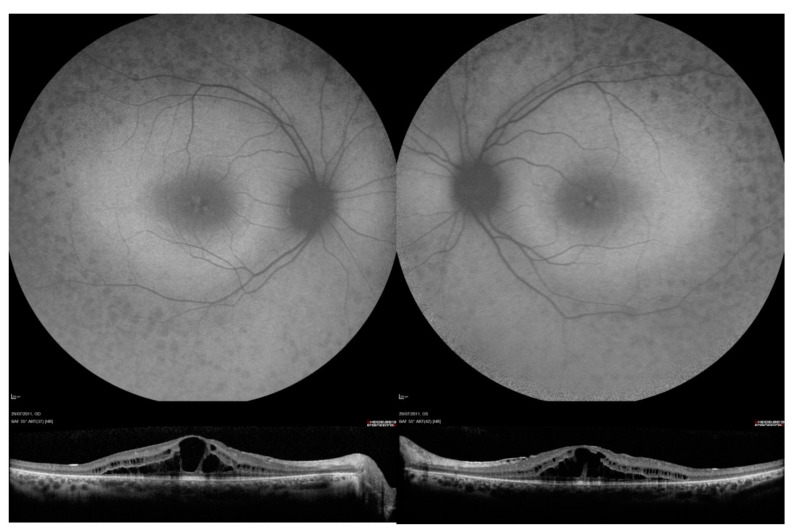
Fundus autofluorescence (FAF) and optical coherence tomography OCT imaging in patient 41, who was compound heterozygous for *USH2A* variants c.2299del and c.8740C > T, p.(Arg2914*). FAF images showed decreased AF signal in the mid-peripheral retinal, raised macular AF and speckled area of raised foveal AF. OCT images show bilateral central cystoid macular oedema.

Retinal images were assessed by M.S and H.F. FAF images were classified using the three patterns described by Fakin et al. [19]. Briefly, the three patterns were: an annulus of increased autofluorescence (AF) signal surrounding the fovea, a focal increase in AF signal at the fovea, or decreased AF signal at the fovea signifying foveal atrophy (Figure 1). SD-OCT images were evaluated for the presence of cystoid macular oedema (Figure 2), which was defined as small hyporeflective lacunae with well-defined boundaries on at least two consecutive B-scans in the macular area [20]. Central retinal thickness was recorded as the average retinal thickness in the central 1 mm of the ETDRS grid (centred on the fovea) and was recorded using Heidelberg Eye Explorer (Heidelberg, Heidelberg Engineering, Germany) [21,22].

### 2.2. Genetic Analysis

Genetic testing was performed at the Oxford Medical Genetics Laboratories, Oxford University Hospitals NHS Foundation Trust, as described previously [23]. Panel-based sequencing was used at the time of writing this publication and the samples were prepared using a customised Agilent’s HaloPlex™ Target Enrichment system kit (Agilent Technologies) designed to capture the coding exons and at least 10bp of the flanking introns of 83 syndromic retinal genes in the Oxford next-generation sequencing IRD phenotype based panel. HaloPlex reactions were prepared as per the manufacturer’s instructions. Libraries were pooled into batches of 14 and sequenced on an Illumina MiSeq instrument (Illumina, San Diego, CA, USA) using a MiSeq v3 kit as per the manufacturer’s instructions. Reads were aligned using Burrows-Wheeler Alignment (BWA) [24] and variants called using Platypus [25]. All findings were validated by Sanger sequencing. Dosage analysis of *USH2A* was performed using MLPA (Probe mix P361-A1and P361-A2, MRC-Holland) with data analysis performed in Coffalyser (MRC-Holland) [26]. The retrospective nature of this study means that some patients were not screened using the syndromic RP panel and were screened using the most appropriate technique available at the time of their presentation.

In silico analysis using three different prediction methods were used to determine the deleteriousness of the variants, Polyphen2 (available at http://genetics.bwh.harvard.edu/pph2/, (accessed on 10 June 2022), Sorting Intolerant from Tolerance (SIFT) (available at http://sift.jcvi.org/, (accessed on 10 June 2022), and Mutation Taster (available at http://www.mutationtaster.org/, (accessed on 10 June 2022), was carried out on all variants identified. The pathogenicity of the variants was graded using the American College of Medical Genetics Criteria [27]. For the purposes of this study, patients were considered to have a confirmed molecular diagnosis if they carried two C5/C4 variants or carried one C5/C4 variant and a C3 variant.

Based on the hypothesis that the severity of the clinical phenotype is correlated with the remaining function of the protein, a strategy to classify patients into groups based on the severity of their genotype was used. A previously described classification that was used in the ProgStar study for the classification of the genotype severity in *ABCA4* retinopathies was used as the basis of the three genotype groups in this study [28]. Genotype group A included patients with two or more severe or null variants, genotype group B included patients with one severe/null variant and at least one missense or in-frame deletion/insertion and genotype group C included patients with at least two missense or in-frame insertion/deletion variants.

### 2.3. Statistical Analysis

Statistical analysis was carried out using Python. Right eyes were used for comparison of visual acuity, FAF pattern and central retinal thickness between groups. A patient was considered to have cystoid macular oedema (CMO) if it was identified in either eye. ANOVA and *t*-test were used to compare continuous variables and Fisher’s exact test was used to analyse contingency tables. A *p*-value of less than 0.05 was considered to be statistically significant.

## 3. Results

In this retrospective study, 72 patients with a molecularly confirmed diagnosis of either USH or NS-ARRP associated with genes implicated in USH syndromes were identified. Of the 72 patients, 39 had a USH phenotype and 33 patients had an NS-ARRP phenotype (Supplementary Tables S1–S3). Of the USH patients, the causative gene was *USH2A* in 28 (Supplementary Table S1), *MYO7A* in 4, *CDH23* in 2, *USH1C* in 2, *GPR98*/*VLGR1* in 2, and *PCDH15* in 1 patient (Supplementary Table S3). All 33 patients with NS-ARRP had variants in *USH2A* (Supplementary Table S2). In addition to these 72 patients, there were 6 patients that were excluded from this study, 3 had variants in other IRD genes, and 3 did not meet the criteria for a molecularly confirmed diagnosis of USH2A because they had 2 class 3 variants.

Eighteen novel variants in 4 genes (13 in *USH2A*, 2 in *GPR98*, 1 in *PCDH15* and 2 in *USH1C*) were identified in 19 patients in this cohort (Table 1 and Table 2, highlighted by *). Of these, 14 patients had USH and 5 patients had NS-ARRP. The in silico analysis and ACMG classification for these variants are summarised in Supplementary Table S4.

### 3.1. Patients with USH2A Associated Disease

*USH2A* was the commonest gene associated with USH and NS-ARRP in this patient cohort and was investigated further to identify genotype-phenotype correlations. Twenty-eight patients with USH and 33 patients with NS-ARRP and a molecularly confirmed diagnosis of *USH2A* were identified.

### 3.2. Phenotype Correlations

Patients with USH, whatever the associated gene, tended to have an earlier onset of retinal disease (other than GPR98/VLGR1) when compared to patients presenting with NS-ARRP. The *USH2A* group, which was analysed in more depth, showed that patients with USH experienced visual symptoms at an earlier age (mean 17.9, SD 12.195 years) compared to patients with NS-ARRP (mean 31.7 years, SD 16.528 years; *p* < 0.001). However, there was no significant difference in BCVA between these two groups (*p* = 0.702) at baseline presentation. The retinal FAF pattern differed between patients with USH and NS-ARRP (*p* = 0.040); a focal discrete increased AF signal at the fovea was noted more often in USH patients (40.9% vs. 12.0%) (Table 1). Cystoid macular oedema was also more commonly identified in patients with USH (*p* = 0.021), but there was no statistical difference in central retinal thickness between the two groups (*p* = 0.183). All patients with USH had hearing loss. Eleven patients (33.3%) with NS-ARRP self-reported mild late-onset hearing loss as the disease progressed, but this was subsequent to their initial presentation at which they were diagnosed with NS-ARRP. Ten patients with USH and 10 patients with NS-ARRP had preservation of ≥10 degrees of the visual field, 1 patient with USH and 3 patients with NS-ARRP had <10 degrees of the visual field, and 1 patient with NS-ARRP had <10 degrees of visual field and a central scotoma. Electrodiagnostic testing was only available for 14 patients with USH2A retinopathy and of these 7 patients had a clinical diagnosis of Usher syndrome and 7 patients had non-syndromic RP. Three individuals from both groups had all, whereas, in the left eye, 5 of the syndromic eyes were unmeasurable, but only 1 of the non-syndromic eyes were unmeasurable for all stimuli. Out of the remaining syndromic eyes, the pattern electroretinogram (pERG) consistently had a delayed peak time and reduced amplitude. Likewise, for the full field electroretinogram (ffERG), where a response was visible, it was severely reduced with a delayed peak time. In the non-syndromic cohort, there were more eyes with measurable responses. The same patterns were observed with severely reduced amplitudes and delayed peak times for all stimuli.

### 3.3. Genotype-Phenotype Correlations

Of the 28 patients diagnosed with USH, 16 (57.1%) had a group A genotype, 11 (39.2%) had a group B genotype and 1 (3.6%) had a group C genotype (Table 2). Of the 33 patients diagnosed with NS-ARRP, 1 (3.0%) had a group A genotype, 15 (45.5%) had a group B genotype and 17 (51.5%) had a group C genotype (Table 2). Genotype groupings differed significantly between patients with USH and NS-ARRP (*p* < 0.001). Patients with a group A genotype had a mean age of first ocular symptoms of 15.5 years compared to 25.1 years for those with a group B genotype and 35.2 years for those with a group C genotype (*p* = 0.004). There was no statistically significant difference in visual acuity, FAF pattern, the presence of cystoid macular oedema or central retinal thickness between the different genotype groups (Table 2).

## 4. Discussion

In this study, we present a cohort of 72 patients with USH and NS-ARRP with a molecularly confirmed diagnosis in genes associated with USH. In our cohort, variants in *USH2A* were most frequently identified than other USH-associated genes. In the *USH2A* group of 61 patients, those with USH showed a more severe retinal phenotype compared to patients with NS-ARRP. Those patients with a more severe genotype were more likely to be diagnosed with USH compared to NS-ARRP and developed ocular symptoms at an earlier age. Indeed, patients with USH presented with ocular symptoms on average 13.8 years earlier than those with NS-ARRP (17.9 vs. 31.7 years, *p* < 0.001). This is in keeping with previous reports that show patients with Usher syndrome type 2A develop symptoms at a younger age compared to patients with NS-ARRP associated with *USH2A* mutations (median age, 15 years vs. 25 years; *p* < 0.001) [3]. The mean age of first ocular symptoms in patients with USH associated with *USH2A* in our cohort is similar to that reported in the literature [1,3,19,29] and the recent RUSH2A study also found that the age of symptom onset in NS-ARRP patients was 31.8 years compared to 18.4 years in USH2A patients [12].

No statistically significant difference in visual acuity was identified between patients with USH and NS-ARRP associated with *USH2A* in this study (*p* = 0.702). The exclusion of descriptive visual acuity values of counting fingers or less from the analysis will have affected these results. Pierrache et al. reported that patients with NS-ARRP achieved the criteria of visual impairment 18 and 13 years later based on visual acuity and visual fields, respectively, when compared to patients with USH2 [3]. Colombo et al. similarly reported worse visual acuities in patients with syndromic *USH2A* and visual acuity was also worse in patients with more severe *USH2A* variants [30]. The RUSH2A trial also reported that their *USH2A* patients with NS-ARRP had greater preservation of visual fields compared to patients with USH after adjustment of disease duration and age of enrolment [12].

FAF images differed significantly between patients with USH and NS-ARRP in this cohort (*p* = 0.040). An increased AF annulus signal around the fovea occurred more frequently in patients with NS-ARRP (72.0% vs. 54.6%), while a focal discrete high AF signal located at the fovea occurred more frequently in patients with USH (40.9% vs. 12.0%). These FAF imaging features suggest that patients with USH had a more advanced retinal disease. Fakin et al. proposed that the discrete focal increase in AF signal at the fovea is a hallmark of foveal involvement and loss of central visual function [19]. These FAF imaging features were also identified by Lenassi et al., who identified an increased AF signal annulus in 39/48 (81.3%) eyes and a discrete focal increase in AF signal in 5/48 (10.4%) eyes in patients with NS-ARRP associated with *USH2A* [9]. These observations are similar to the findings in our cohort.

Patients with USH associated with *USH2A* were more likely to have cystoid macular oedema than patients with NS-ARRP associated with *USH2A* in our cohort (38.1% vs. 4.8%, *p* = 0.021). The occurrence of cystoid macular oedema in USH reported in the literature is highly variable and has been reported in between 14.2% and 56% of patients [19,20,31].

A comparison of the *USH2A* genetic variants identified in patients with USH and NS-ARRP in our cohort showed that all but one of the patients that had at least two null/truncating variants had syndromic features. We propose that patients with severe variants, which produce a truncated protein that is predicted to undergo nonsense-mediated decay, will develop a more severe phenotype that includes hearing loss due to a very low amount of (or no) functioning protein, which is in keeping with Pierrache et al.’s findings [3]. Milder variants may produce a protein that retains enough function to produce an NS-ARRP phenotype or the development of hearing loss later in life, depending on any modifying or environmental factors. Inaba et al. recently reported that all patients with USH (*USH2A* associated) and with severe retinal phenotypes had variants that produce a truncated protein when compared to patients with NS-ARRP [15]. Hartel et al. [14] and Lee et al. [32] reported that the presence of two truncating *USH2A* variants was associated with more severe and progressive hearing impairment compared with either one truncating variant and one non-truncating variant or only non-truncating variants. Molina-Ramirez et al. also reported that truncating *USH2A* variants were more frequently identified in patients with hearing loss [33]. Sengilo et al. used electrodiagnostic testing to show that patients with Usher syndrome type 2 had reduced cone function compared to patients with NS-ARRP and also reported that patients with USH tended to have more severe *USH2A* variants [2]. Lenassi et al. proposed that NS-ARRP associated with *USH2A* was associated with the presence of at least one retinal disease-specific *USH2A* variant [9]. They also reported that null variants were rare in patients with NS-ARRP and were more common in patients with USH and proposed that the retinal-specific variants may result in some functional protein that results in normal cochlear development [9]. The RUSH2A study reported that truncating variants were significantly associated with the USH phenotype. In the RUSH2A study, all 42 patients with two truncating variants had a USH2 phenotype and these patients accounted for 53% of their USH2 cohort. They found that the c.2299delG, p.(Glu767SerfsTer21) variant was enriched in patients with USH whilst the c.2276G > T, p.(Cys759Phe) variant was enriched in the NS-ARRP patients and that the overall allele frequency of missense variants was also higher in patients with NS-ARRP [12]. The authors proposed that there is a dose-related effect on hearing loss and that missense variants occurring within the interfibronectin domain were hypomorphic and were associated with NS-ARRP.

In our cohort, 11 (33.3%) of the NS-ARRP group self-reported later onset hearing loss; this occurred subsequent to their initial presentation. It is possible that the hearing impairment in some of the older patients of this group may be due to natural ageing processes. However, later onset hearing loss in patients with *USH2A* variants who have initially presented with NS-ARRP was first reported by Rivolta et al., who found that this was significantly associated with the c.2299delG, p.(Glu767SerfsTer21) variant in their cohort and proposed that subjective late-onset hearing loss can occur in NS-ARRP secondary to *USH2A* variants [11]. Indeed, the mean age of NS-ARRP patients in our cohort was 48.7 years and the 2021 World Health Organisation world report on hearing reported the global prevalence of moderate or higher grade hearing loss was 3.9% in patients aged between 45-49 years [34].

Blanco-Kelly similarly reported late-onset mild to moderate hearing loss in NS-ARRP patients, which was associated with the c.2276G > T, p.(Cys759Phe) variant [1]. This variant was detected in 4/11 (36.4%) of our patients with late-onset hearing loss but also in 10 NS-ARRP patients who had not reported issues with hearing at the time of this study. It is important to ensure that appropriate genetic counselling is carried out prior to genetic testing, as for those originally diagnosed with NS-ARRP, it should be explained that *USH2A* variants may be associated with later onset hearing impairment. In addition, those patients with NS-ARRP secondary to *USH2A* variants who report symptoms of late-onset hearing loss may benefit from formal audiology testing and hearing aids to address the impact of dual sensory impairment and improve quality of life.

## 5. Conclusions

This study has shown that the retinal phenotype of patients with USH is more severe with an earlier age of symptom onset, with FAF central involvement, and a higher occurrence of cystoid macular oedema compared to the NSRP cohort. Analysis of genetic variants in *USH2A* showed that patients with a more severe genotype were more likely to be diagnosed with USH compared to NS-ARRP. We have reported 18 novel variants in genes associated with Usher syndrome, thus expanding the genetic spectrum of known pathogenic variants. This information is important in providing an accurate molecular diagnosis to affected patients. This is becoming increasingly relevant in the context of current clinical trials and potential therapies for Usher-related genes.

## Figures and Tables

**Table 1:** USH vs. NS-ARRP with USH2A.

	USH		Non-Syndromic RP		*p* Value
	Mean	*n*	Mean	***n***	
Age at first ocular symptoms (yrs)	17.9 (SD 12.2)	26	31.7 (SD 16.5)	32	<0.001
Age at assessment (yrs)	39.4 (SD 11.8)	29	48.7 (SD 15.6)	33	0.011
LogMAR visual acuity *	0.35 (SD 0.29)	27	0.32 (SD 0.39)	31	0.702
FAF pattern *	*n* = 22		*n* = 25		
Hyperautofluorescent ring	12 (54.6%)		18 (72.0%)		0.040
Hyperautofluorescent foveal patch	9 (40.9%)		3 (12.0%)		
Foveal atrophy	0		2 (8.0%)		
OCT findings					
CMO	8 (38.1%)	21	1 (4.8%)	21	0.021
Central retinal thickness (µm) *	274.5 (SD 113.2)	21	237.4 (SD 54.3)	21	0.183

* Right eyes were used to compare visual acuity, FAF patterns and central retinal thickness between the groups. There was no difference in visual acuity (*p* = 0.986). FAF pattern (*p* = 1.0) or central retinal thickness (NS-ARRP *p* = 0.704. USH *p* = 0.895) between right and left eyes. A patient was considered to have CMO if it was identified in either eye. CMO = cystoid macular oedema.

**Table 2:** Comparison between phenotypic severities between genotype groups of USH2A.

	Genotype A		Genotype B		Genotype C		*p* Value
	Mean	*n*	Mean	*n*	Mean	*n*	
Syndromic *USH2A*		16		10		1	<0.001
NS-ARRP		1		16		17	
Age at first ocular symptoms (yrs)	15.5 (SD 5.4)	12	25.1 (SD 16.1)	26	35.2 (SD 15.2)	16	0.004
Age at assessment (yrs)	39.3 (SD 13.7)	17	42.9 (SD 13.4)	26	52.4 (SD 12.9)	18	0.016
LogMAR * visual acuity	0.40 (SD 0.31)	16	0.34 (SD 0.38)	25	0.27 (SD 0.30)	16	0.609
FAF pattern *	*n* = 15		*n* =17		*n* = 15		
Hyperautofluorescent ring	8 (53.3%)		14 (82.4%)		10 (66.7%)		0.411
Hyperautofluorescent foveal patch	6 (40%)		3 (17.7%)		4 (26.7%)		
Foveal atrophy	1 (6.7%)		0		1 (6.7%)		
OCT findings							
CMO	4 (33.3%)	12	4 (21.1%)	19	0	13	0.078
Central retinal thickness (µm) *	283.8 (SD 130.1)	13	247.2 (SD 60.8)	18	232.0 (SD 40.9)	13	0.299

* Right eyes were used to compare visual acuity, FAF patterns and central retinal thickness between the groups. A patient was considered to have CMO if it was identified in either eye. CMO = cystoid macular oedema.

## Data Availability

Not applicable.

## Supplementary Materials

The following supporting information can be downloaded at: https://www.mdpi.com/article/10.3390/genes13081423/s1, Table S1: Genetic and phenotypic characteristics of patients USH with *USH2A* variants. Table S2: Genetic and phenotypic characteristics of patients with NS-ARRP with *USH2A* variants. Table S3: Genetic and phenotypic characteristics of patients with genes implicated in USH syndromes other than *USH2A.* Table S4: Summary of analysis of novel variants identified in this study
