# Supplementary material for: Phenotypic and Genetic Characteristics in a Cohort of Patients with Usher Genes
Source: Genes (Basel). 2022 Aug 10;13(8):1423. doi: 10.3390/genes13081423 (PMC9407802; doi:10.3390/genes13081423)
Supplement: Supplementary file 1 [file genes-13-01423-s001.zip › genes-1827009-supplementary.pdf]

ID	Age at baseline examination	Sex	VA logMAR OD	VA logMAR OS	Visual field	Presenting symptoms	Age of onset	Hearing impairment	Variants		Geno type
1	32	F	0.6	0.6	<10°	Nyctalopia + Visual field constriction	24	Y	c.2276G>T p.(Cys759Phe)	c.12294+2T>A† & c.12295-2A>G	A
2	27	M	0.1	0.22	>10° + peripheral islands	Nyctalopia + Visual field constriction	Late Teens	Y	del exon 27	c.10561T>C p.(Trp3521Arg)	B
3	33	M	0.18	0.18	NP	Nyctalopia	12	Y	c.7595-3C>G	c.1256G>T p.(Cys419Phe)	B
4	35	F	0.18	0.18	>10° + peripheral temporal island	Nyctalopia	6	Y	c.6862G>T p.(Glu2288*)	exon 40 del	A
5	64	F	0.3	0.18	>10° . central scotoma and relatively preserved peripheral fields	Nyctalopia + Visual field constriction	63	Y	c.2299del	c.13316C>T p.(The4439Ile)	B
6	41	F	0.18	0.3	NP	Nyctalopia + Visual field constriction	Teens/ childhoo d	Y	c.2299del	c.2299del	A
8	51	F	HM	HM	NP	Nyctalopia	13.5	Y	c.1606T>C p.(Cys536Arg)	c.1256G>T p.(Cys419Phe)	C
9	42	F	0.48	0.3	10° in both eyes. Left eye has a small temporal peripheral island	Nyctalopia	16	Y	c.2299delG	c. 11864G>A p.(Trp3955*)	A
10	66	M	0.78	PL	NP	Nyctalopia	15	Y	c.2299delG p.(Glu767fs)	c.2299delG p.(Glu767fs)	A

11	26	M	0.18	0.18	>10° centrally but constricted peripheral fields	Nyctalopia	24	Y	c.2299del	c.12295-2A>G	A
12	44	M	HM +	0.1	>10° centrally surrounded by a central scotoma. Relatively intact peripheral fields (OS)	Nyctalopia and Dark adaptation	37	Y	c.7932G>A p.(Trp2644*)	c.13331C>T p.(Pro4444Leu)& c.6364G>T p.(Ala2122Ser)	B
13	34	F	0.48	0.48	>10° and relatively intact peripheral fields	Nyctalopia	10	Y	c.14407_14420delinsTCA p.(Ile4803fs)*	c.14407_14420delinsTCA p.(Ile4803fs) †	A
15 #	41	M	0.6	0.6	NP	Nyctalopia	15	Y	c.9583G>T p.(Gly3195*)†	c.1859G>T p.(Cys620Phe)	B
16 #	39	M	0.6	0.6	NP	Nyctalopia	Teens	Y	c.9583G>T p.(Gly3195*)†	c.1859G>T p.(Cys620Phe)	B
18	26	M	0	0.22	NP	Nyctalopia	17	Y	c.7595-3C>G	c.12697_12698del p.(Trp4233fs)	A
19	48	M	0.3	0.3	10°	Nyctalopia + Dark adaptation	7.5	Y	c.6539delT p.(Met2180fs) †	c.4133T>C p.(Leu1378Pro)	B
20	41	F	0.7	0.6	<10°	Nyctalopia	11	Y	c.3420_3423delTTAC	c.5528C>T p.(Pro1843Leu)	B
23	57	M	0	0	NP	Nyctalopia + Visual field constriction	20ies	Y	c.2299del	c.6925T>C p.(Cys2309Arg)	B
27	30	M	0.09	0	NP	Nyctalopia	16	Y	c.5899_5900del	del of exons 22-24	A

32	45	F	0.48	0.3	NP			Y	c.5614delins12	c.14803C>T p.(Arg4935*)	A
33	40	M	0.3	0.48	10 ° with a small peripheral island of vision			Y	c.2299del	c.2299del	A
35	55	F	1	0.78	NP	Nyctalopia	11	Y	c.9469C>T p.(Gln3157*)	c.10586-1_10595delins13†	A
39	36	M	0	0	NP	Visual field constriction	34	Y	c.5899_5900delAA	c.4732C>T p.(Arg1578Cys)	B
40	43	M	1	1	NP	Colour vision abnormalities	Teens	Y	c.2299del p.(Glu767fs)	c.1550+1G>T†	A
41	22	M	0.1	0.1	NP	Nyctalopia + Dark adaptation	21	Y	c.2299del	c.8740C>T p.(Arg2914*)	A
43	39	M	0.3	0.48	NP	Nyctalopia	16	Y	c.13374delA p.(Glu4458fs)	USH2A dup exons 63-64†	A
139	21	F	0.18	0.18	NP			Y	c.2299del	c.7595-21444A>G	A
157	42	M	0.18	0.18	Central 10° preserved with an adjacent central scotoma. Constricted peripheral fields	Nyctalopia. constricted visual fields	30	Y	c.100C>T p.(Arg34*)	c.1381C>T p.(Pro461Ser) † & c.6118T>G p.(Cys2040Gly) c.7475C>T p.(Ser2492Leu)	B

Supplementary Table S1. Genetic and phenotypic characteristics of patients USH with *USH2A* variants.
Key: novel variants indicated by†. Abbreviations: approximately (~), Male/Female (M/F); Yes /No (Y/N); count fingers (CF); hand motions (HM); siblings (#)

ID	Age at baseline examination	Sex	VA logMAR OD	VA logMAR OS	Visual field	Presenting symptom	Age of onset	Hearing impairment	Variants		Geno type
17	59	F	0.95	1.18	NP	Nyctalopia	Teens	Y	c.14343+1G>C	c.3407G>A p.(Ser1136Asn)	B
37	71	M	HM	HM	NP	Nyctalopia	Late 40s	Y	c.2299delG	c.5614delins12 p.(Ala1872Leufs)	A
45	69	F	0.18	0.18	NP	Nyctalopia + Visual field constriction	53	N	c.1606T>C p.(Cys536Arg)	c.7525C>T p.(Arg2509Trp)	C
47	61	F	PL	NPL	NP	Nyctalopia	34	N	c.2276G>T p.(Cys759Phe)	c.3407G>A p.(Ser1136Asn)	C
49	45	F	0	0.18	NP	Nyctalopia + Photosensitivity	32	Y	c.2276G>T p.(Cys759Phe)	c.12739G>A p.(Gly4247Arg) †	C
50	35	M	0	-0.2	<10 ° central fields with peripheral islands of vision	Nyctalopia	24	N	c.2276G>T. p.(Cys759Phe)	c.11875_11876delCA	B
51	57	F	0	0.1	~10° central field with peripheral islands of vision	Nyctalopia + Visual field constriction	52	N	c.2276G>T p.(Cys759Phe)	c.10073G>A p.(Cys3358Tyr)	C
52	43	M	0.6	0.3	>10° central field	Nyctalopia	Childh ood	N	c.2299delG. p.(Glu767fs)	c.2276G>T p.(Cys759Phe)	B
54	33	F	0.1	0.1	>10° with central scotomas and minimally constricted peripheral fields	Visual field constriction	34	N	c.920_923dup p.(His308*)	c.10073G>A p.(Cys3358Tyr)	B
57	47	M	0.1	0	Slightly >10° central fields	Dark adaptation	27	N	c.13316C>T p.(Thr4439Ile)	c.12574C>T p.(Arg4192Cys)	C

61	68	M	0.18	0	>10° with central scotomas and minimally constricted peripheral fields	Visual field constriction	65	N	c.2276G>T p.(Cys759Phe)	c.8088T>A p.(Tyr2696*)†	B
66	46	F	1.6	HM	NP	Nyctalopia	15	Y	c.13374delA p.(Glu4458fs)	c.2276G>T p.(Cys759Phe)	B
83	67	F	0.3	0.3	NP	Visual field constriction	73	Y	c.10342G>A p.(Glu3448Lys)	c.11858G>A p.(Ser3953Asn) † and c.6670G>T p.(Gly2224Cys)	C
87	56	F	0.48	0.48	NP	Visual field constriction	25	Y	c.11709C>G p.(Tyr3903*)†	c.12874A>G p.(Asn4292Asp)	B
92	57	F	1	HM	NP	Nyctalopia	35	N	c.10342G>A p.(Glu3448Lys)	c.10342G>A p.(Glu3448Lys)	C
96	69	M	0.1	CF	~ 10°	Photosensitivity	61	N	Exon 1 (non-coding) and exon 2 deletion*	c.10073G>A p.(Cys3358Tyr)	B
98	34	M	0	0	~ central 10° field with peripheral islands of vision	Visual field constriction	19	Y	c.2299del	c.10073G>A p.(Cys3358Tyr)	B
99	28	F	-0.08	-0.08	NP	Nyctalopia + Visual field constriction	27	N	c.2276G>T p.(Cys759Phe)	c.8740C>T p.(Arg2914*)	B
101	60	M	0.1	0.1	>10° and central scotoma		Teens	Y	c.2276G>T p.(Cys759Phe)	c.3407G>A p.(Ser1136Asn)	C
102	46	M	0.18	0.18	Central scotoma and constricted fields	Nyctalopia	42	N	c.11156G>T p.(Arg3719Leu)	c.11156G>T p.(Arg3719Leu)	C

104	22	F	0	0	<10 ° surrounded by central scotoma. mild constriction of the peripheral fields.	Nyctalopia	19	N	c.2276G>T p.(Cys759Phe)	c.920_923dup p.(His308*)	B
110	26	F	0.09	0	<10° surrounded by central scotoma. peripheral islands of vision to I4e and mild constriction of V4e	Nyctalopia + Visual field constriction	24	N	c.12574C>T p.(Arg4192Cys)	c.1859G>T p.(Cys620Phe)	C
115	53	F	0.78	0.6	NP	Nyctalopia	41	N	c.1618C>T p.(Gln540*)	c.10342G>A p.(Glu3448Lys)	B
116	31	F	0.18	0.18	NP	Nyctalopia	18	N	c.10073G>A p.(Cys3358Tyr)	c.10561T>C p.(Trp3521Arg)	C
117	35	M	0.3	0.3	<10°		21	Y	c.2276G>T p.(Cys759Phe)	c.802G>C p.(Gly268Arg)	C
123	70	M	0.78	1	NP	Nyctalopia + Reduced VA or blurring	50	N	c.2276G>T p.(Cys750Phe)	c.12574C>T p.(Arg4192Cys)	C
124	20	M	0.18	0	>10° central field (to I4e) and the peripheral fields are mildly constricted	Nyctalopia	17	Y	c.12574C>T p.(Arg4192Cys)	c.11874_11875delCA	B
127	71	F	0.78	0.78	NP	Nyctalopia	38	N	c.10073G>A p.(Cys3358Tyr)	c.7475C>T	C

										p.(Ser2492Leu); c.6118T>G p.(Cys2040Gly)	
128	28	M	0.18	0	NP	Nyctalopia	16	N	c.2276G>T p.(Cys759Phe)	c.2299del	B
129	55	F	0.1	0	>10° but constricted peripheral fields (L>R)	Nyctalopia	25	N	c.2276G>T p.(Cys759Phe)	c.9346C>A p.(Pro3116Thr)	C
147	50	F	0.4	0.6	NP	Nyctalopia	13	Y	c.2299delG	c.10342G>A p.(Glu3448Lys)	B
154	44	M	-0.02	-0.02	>10° of central field surrounded by central scotoma. Relitavly intact peripheral fields	Photophobia. (constricted fields)	44	N	c.10073G>A p.(Cys3358Tyr)	c.12574C>T p.(Arg4192Cys)	C
159	52	M	0.3	0.18	<10°	Nyctalopia (26) + constricted visual fields (30's) + reduced VA (50's)	25 (mid 20s)	N	c.1214del p.(Asn405fs)	c.10073G>A p.(Cys3358Tyr)	C

Supplementary Table S2. Genetic and phenotypic characteristics of patients with NS-ARRP with *USH2A* variants.
Key: novel variants indicated by†. Abbreviations: approximately (~); Male/Female (M/F); Yes /No (Y/N); count fingers (CF); hand motions (HM)

ID	Age at baseline examination	Sex	VA logMAR OD	VA logMAR OS	Visual fields	Presenting symptom	Age of onset	Hearing impairment	Gene	Variants	
21	13	F	0.3	0.18	Relatively intact visual fields	Nyctalopia	11	Y	MYO7A	c.3508G>A p.(Glu1170Lys)	c.4951G>A p.(Gly1651Ser)
22	7	M	0.18	PL	NP	Reduced VA	7	Y	CDH23	c.3880C>T p.(Gln1294*)	c.3880C>T p.(Gln1294*)
24	52	F	1	HM	NP			Y	PCDH15	c.173-2A>T†	c.1751dup p.(Tyr584*)
28	8	M	0.3	0.18	Constricted fields >10° to I4e and mildly constricted peripheral fields	Nyctalopia	7.5	Y	CDH23	c.1112delT p.(Ile371*)	c.7362G>A p.(Thr2454Thr)
29	11	F	0.1	0.1	~ 10° field for I4e and relatively intact peripheral fields to V4e	Nyctalopia	Lifelong	Y	USH1C	c.1085+2T>C*	c.238dup p.(Arg80*)
31	56	M	0.9	0.8	<10°	Nyctalopia	10	Y	USH1C	c.1039C>T p.(Gln347*)	c.658C>T p.(Arg220*)†
34	33	M	0	0	NP	Nyctalopia	24	Y	GPR98/VLGR1	c.1603C>T p.(Arg535*)†	c.1603C>T p.(Arg535*)†
42	59	F	PL	PL	NP	Reduced VA or blurring	20ies	Y	GPR98/VLGR1	c.16111del p.(Ser537*) †	Exon 21 deletion
143	4	M	0.15	0.15	NP			Y	MYO7A	c.397C>A p.(His133Asn)	c.3892G>A p.(Gly1298Arg)

161	5	F	0.2	0.22	Constriction of the superior peripheral field in the right eye but otherwise relatively intact peripheral fields. <10° to I4e.	Nyctalopia + constricted visual fields	5	Y	MYO7A	c.1662dup p.(Ala555*)	c.4117C>T p.(Arg1373*)
162	7	F	0.12	0.14	Constriction of the superior peripheral field in the right eye but otherwise relatively intact peripheral fields. <10° to I4e in the left eye.	Nyctalopia + constricted visual fields	7	Y	MYO7A	c.1662dup p.(Ala555*)	c.4117C>T p.(Arg1373*)

Supplementary Table S3. Genetic and phenotypic characteristics of patients with genes implicated in USH syndromes other than *USH2A.*
Key: novel variants indicated by†. Abbreviations: approximately (~); Male/Female (M/F); Yes /No (Y/N); count fingers (CF); hand motions (HM)

Gene	Variant	Variant type	SIFT	Poly-Phen2	MutationTaster	SpliceAI splicing defect	ACMG class
USH2A	dup exons 63-64	Structural variant	-	-	-	-	5
USH2A	Exon 1 (non-coding) and exon 2 deletion	Structural variant	-	-	-	-	5
USH2A	c.1381C>T p.(Pro461Ser)	Missense	Deleterious	Probably damaging (HumDiv)<br><br>Possible damaging (HumVar)	Disease causing	No	3
USH2A	c.1550+1G>T	Splice site	-	-	-	Yes	5
USH2A	c.6539delT p.(Met2180fs)	Nonsense	-	-	-	No	5
USH2A	c.8088T>A p.(Tyr2696*)	Nonsense	-	-	Disease causing	No	5
USH2A	c.9583G>T p.(Gly3195*)	Nonsense	-	-	Disease causing	No	5
USH2A	c.10586-1_10595delins13	Nonsense	-	-		-	5
USH2A	c.11709C>G p.(Tyr3903*)	Nonsense	-	-	Disease causing	No	5
USH2A	c.11858G>A p.(Ser3953Asn)	Missense	Deleterious	Probably damaging	Disease causing	No	3
USH2A	c.12294+2T>A	Splice site	-	-	-	Yes	5
USH2A	c.12739G>A p.(Gly4247Arg)	Missense	Deleterious	Possibly damaging (HumDiv)<br><br>Benign (HumVar)	Disease causing	No	3
USH2A	c.14407_14420delinsTCA p.(Ile4803fs)	Nonsense	-	-	-	-	5
USH1C	c.658C>T p.(Arg220*)	Nonsense	-	-	Disease causing	No	5
USH1C	c.1085+2T>C	Splice site	-	-	-	Yes	5

PCDH15	c.173-2A>T	Splice site	-	-	-	Yes	5
GPR98/VLGR1	c.16111del p.(Ser537*)	Nonsense	-	-	-	No	5
GPR98/VLGR1	c.1603C>T p.(Arg535*)	Nonsense	-	-	Disease causing	No	5

Supplementary Table S4. Summary of analysis of novel variants identified in this study
